# Advancing functional and translational microbiome research using meta-omics approaches

**DOI:** 10.1186/s40168-019-0767-6

**Published:** 2019-12-06

**Authors:** Xu Zhang, Leyuan Li, James Butcher, Alain Stintzi, Daniel Figeys

**Affiliations:** Ottawa Institute of Systems Biology and Department of Biochemistry, Microbiology and Immunology, Faculty of Medicine, University of Ottawa, Ottawa, Ontario Canada

**Keywords:** Drug-microbiome interactions, Host-microbiome interactions, Meta-omics, Microbiome, Microbiome assay, Multi-omics, Personalized medicine

## Abstract

The gut microbiome has emerged as an important factor affecting human health and disease. The recent development of –omics approaches, including phylogenetic marker-based microbiome profiling, shotgun metagenomics, metatranscriptomics, metaproteomics, and metabolomics, has enabled efficient characterization of microbial communities. These techniques can provide strain-level taxonomic resolution of the taxa present in microbiomes, assess the potential functions encoded by the microbial community and quantify the metabolic activities occurring within a complex microbiome. The application of these meta-omics approaches to clinical samples has identified microbial species, metabolic pathways, and metabolites that are associated with the development and treatment of human diseases. These findings have further facilitated microbiome-targeted drug discovery and efforts to improve human health management. Recent in vitro and in vivo investigations have uncovered the presence of extensive drug-microbiome interactions. These interactions have also been shown to be important contributors to the disparate patient responses to treatment that are often observed during disease therapy. Therefore, developing techniques or frameworks that enable rapid screening, detailed evaluation, and accurate prediction of drug/host-microbiome interactions is critically important in the modern era of microbiome research and precision medicine. Here we review the current status of meta-omics techniques, including integrative multi-omics approaches, for characterizing the microbiome’s functionality in the context of health and disease. We also summarize and discuss new frameworks for applying meta-omics approaches and microbiome assays to study drug-microbiome interactions. Lastly, we discuss and exemplify strategies for implementing microbiome-based precision medicines using these meta-omics approaches and high throughput microbiome assays.

## Introduction

The human gut harbors trillions of microbial cells and thousands of different species from diverse phylogenetic backgrounds, including bacteria, archaea, and various microbial eukaryotes [1]. Altogether, this community of microorganisms, termed the gut microbiota, has a similar cell number to that of human cells [2] and 450-fold more genes than the human genome [3]. These gut microbiota genomes, namely the metagenome, encode functions and metabolic pathways that participate in various host biological processes, including metabolism, nutrition, and immunity [4–6]. Given the high complexity of the human gut microbiota and the challenges in culturing a high proportion of gut microbial species [7], most microbiome studies employ “meta-omics” approaches, including 16S rRNA gene sequencing, metagenomics, metatranscriptomics, metaproteomics, and metabolomics, which directly examine the phylogenetic markers, genes, transcripts, proteins, or metabolites from the samples [8].

In the past two decades, meta-omics based research has revealed significant associations between the gut microbiome and human diseases, including obesity, diabetes, inflammatory bowel disease (IBD), cardiovascular disease, and various cancers [4, 5]. Several studies have also demonstrated causative roles for the gut microbiome in inducing or alleviating the development of disease following variants of Koch’s postulates [9–11]. Given that the composition of the human microbiota is highly dynamic and can be altered with drugs or dietary interventions [12], the microbiome has been proposed as a druggable target in humans [13]. Accumulating evidence also supports the idea that many drugs, such as metformin [14, 15], may alleviate disease, at least in part, through modulating the gut microbiome. Recent large-scale screening of > 1000 drugs on the growth of selected gut bacterial species also highlighted the wide impacts of various drugs on individual microbes [16]. In addition, the existence of bidirectional drug-microbiome interactions for many clinically prescribed drugs has been demonstrated to impact drug efficacy and/or toxicity [17, 18]. As medicine is currently pursuing more precise disease treatment and health management, it is vital that the microbiome is fully integrated into future therapeutic strategies [19].

Nevertheless, our understanding on the mechanisms underlying host-microbiome and drug-microbiome interactions is still very limited. Several databases linking specific microbial species/strains or microbial metabolic pathways to specific diseases have been published [20–22]; however, these databases remain incomplete and most clinically prescribed drugs have not been assessed for their impact on the composition and function of human microbiomes. In addition, the composition of the human microbiome differs between individuals and is affected by various factors such as diet, lifestyle, and host genetics [23–26]. Thus, each patient’s microbiome will respond differently to therapeutic treatments, and we currently cannot accurately predict these responses in advance. Fortunately, recent microbiome studies have expanded beyond simply profiling microbiota compositions and are increasingly characterizing microbial functions by using functional meta-omics approaches such as metatranscriptomics and metaproteomics [27–29]. The development and optimization of various in vitro microbiome culturing models, such as HuMiX [30], SHIME [31], and RapidAIM [32], opens the door to rapidly screen drugs against individual microbiomes. Herein we summarize the current development of various functional meta-omics approaches, highlighting efforts to integrate findings across meta-omic platforms and discuss their applications in host-microbiome, drug-microbiome, and microbe-microbe interaction studies at the interface of precision medicine.

## Functional meta-omics approaches for studying the microbiome

**Fig. 1:**
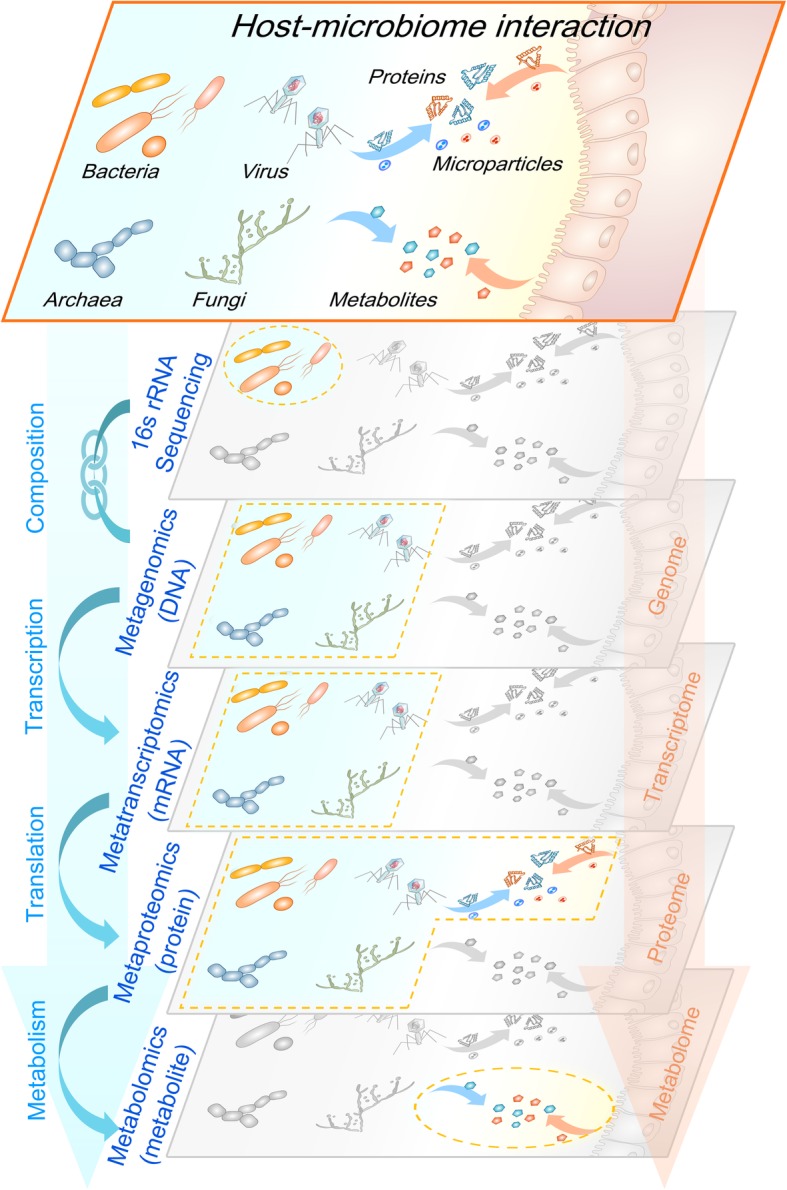
Meta-omics approaches for the study of host-associated microbiomes. Each meta-omics approach reveals different layers of information in the intestinal eco-systems

The human gut consists of host and microbial cells, as well as secreted proteins, metabolites, and microvesicles, all of which may interact with each other to impact human health. Different meta-omic approaches each examine different aspects of this intestinal ecosystem at different levels with their own advantages (detailed in this section) and disadvantages (or challenges discussed in Table 1) (Fig. 1). Technical details on these meta-omics techniques and their associated bioinformatic data processing tools have been reviewed elsewhere [43–47]. Here we focus on the key information that can be obtained from each –omic approach, with a particular focus on those that characterize functional and metabolic activities; namely metatranscriptomics, metaproteomics, and metabolomics.

**Table 1:** Challenges for metatranscriptomics, metaproteomics, and metabolomics in microbiome studies

Metatranscriptomics, metaproteomics, and metabolomics each have their own shortcomings. Metatranscriptomic experiments rely on obtaining sufficient high-quality RNA from the sample source; something which can be quite challenging due to the ubiquitous presence of RNases in host-derived samples. In addition, metatranscriptomic sequencing can often become saturated with reads from less-informative, but highly expressed transcripts (i.e., ribosomal proteins, translation factors, major outer membrane proteins) from the most abundant microbes present, obscuring the detection of functionally important, but less abundant transcripts/proteins. Therefore, the quality of RNA as well as the depth of measurement is important aspects that need to be evaluated or considered in metatranscriptomics.	
Compared to metagenomics and metatranscriptomics, metaproteomics has a lower depth of measurement and can only capture 10–20% of expressed proteins in human gut microbiomes [27, 33, 34]. MS spectra can also be saturated with the highly abundant proteins from dominant species, and this issue is unlikely to be resolved by increasing the speed or time of MS scanning. However, applying off-line protein/peptide separation (such as using sodium dodecyl sulfate polyacrylamide gel electrophoresis) or targeted enrichment strategies (such as using activity-based probes [35]) may to some extent address this limitation. In addition, as metaproteomics is still in its infancy for the study of microbiomes, there is still a lack of universal guidelines and protocols for properly performing metaproteomic experiments and interpreting metaproteomic results. Therefore, careful considerations should be made for sample preparation, MS measurement, bioinformatic workflows, and data reporting (readers are directed to this perspective article for more details [36]).	
The major challenge for metabolomics in microbiome studies is the difficulty to distinguish host- and microbiome-origin metabolites and directly link metabolites to specific taxa [37]. One feasible approach to address this issue is to identify co-variations between metabolites and microbial species, which is indicative for species-specific metabolite production, through integrative analysis of microbiota compositions with metabolite profiles [38–41]. Other approaches, such as protein stable-isotope probing (protein-SIP) [42], can also link the metabolism of a specific substrate to phylogenetic information by monitoring the isotopes in microbial protein sequences with mass spectrometers and may eventually aid in microbiome metabolic reconstructions.

The composition and functional capacity of human microbiomes have been well characterized using next-generation sequencing techniques, such as amplicon sequencing (e.g., 16S rRNA gene) and shotgun metagenomics. In particular, shotgun metagenomics is now widely applied in microbiome studies, providing valuable functional information down to the strain level and for all types of microorganisms (including archaea, fungi, and viruses) [48–52]. More recently, metagenomic sequencing of hundreds to tens of thousands of samples was carried out in large scale projects studying the role of the microbiome in human disease, including studies on early-onset type 1 diabetes (T1D) [53, 54], IBD [55], pre-diabetes [56], and colorectal cancer [57, 58]. In particular, these studies employed longitudinal and/or multi-omic experimental designs, which enabled better characterization of the dynamic changes and functional activities of the microbiome during disease progression. Despite their costs and technical challenges, longitudinal and multi-omic experimental designs are becoming indispensable for unravelling host-microbiome interactions during disease and for assessing causality in clinical microbiome investigations. A beneficial spin-off from these massive metagenomic sequencing projects has been their deposition into easily accessible databases. This has allowed researchers to leverage these datasets to create reference databases for future studies. Examples include a database with > 150,000 microbial reference genomes [59] and a human gut microbial gene catalog database consisting of > 9,800,000 genes [3]. These are valuable resources for functional studies of the human microbiome using metatranscriptomic and metaproteomic approaches.

The presence of a gene does not necessarily mean the gene is expressed. Thus the direct measurement of transcripts or proteins using metatranscriptomics or metaproteomics, respectively, is becoming an important complementary approach for metagenomics. Metatranscriptomics employs similar analytical approaches (e.g., nucleic acid sequencing) as metagenomics. Accordingly, the software tools employed for metagenomics are often adapted for metatranscriptomic data processing [29]. Using the same tools for both metatranscriptomics and metagenomics provides a straightforward route for their integration in microbiome studies [29, 33, 60]. Their combination not only improves microbial genome assembly and gene prediction [33], but can also enable the identification of genes which are induced/repressed under specific conditions. In addition, identifying genomes with active transcription can distinguish metabolically active microbes from inert or dead microbes [33]. In contrast with metagenomics and metatranscriptomics, metaproteomics measures expressed proteins, the basic functional unit for most cellular biological processes, using high-resolution mass spectrometry (MS). Metaproteomics should in principle provide superior insight into gut microbial functionality as compared with metatranscriptomics, since not all transcripts are subsequently translated into proteins. In the past, metaproteomics was rarely employed in gut microbiome studies, at least in part, due to the lack of efficient bioinformatic tools and low protein measurement depth [61]. Fortunately, the recent development of metaproteomic data processing tools, such as MetaLab [62], MetaProteomeAnalyzer [63], and Galaxy-P [64], has greatly advanced our ability to analyze metaproteomic data (readers are directed to extensive reviews for more information [61, 65]). This has enabled deep characterization of microbiome protein compositions, with some reports quantifying > 50,000 unique microbial protein groups in a single study [38, 66]. It is noteworthy that metaproteomics identifies and quantifies proteins from all organisms present within the microbiome, regardless of their phylogenetic origin, and can quantify host proteins as well [27, 33, 67]. This feature is of particular importance when studying host-associated microbiomes in vivo and can uncover important players (e.g., extracellular vesicles [27]) mediating host-microbiome interactions.

In addition to the microbiome’s functional activity, a further goal of microbiome research is to measure metabolic outcomes. Metabolomics directly measures the metabolites present in the intestine using analytical techniques such as nuclear magnetic resonance (NMR) spectroscopy or mass spectrometry (MS). Given the higher sensitivity of MS compared to NMR [68], the field of metabolomics has increasingly shifted to MS-based approaches. Readers are directed to previous reviews for more details on methodologies for fecal metabolomics [69–71]. Fecal water is among the most commonly used sample types for fecal metabolomic studies, although there are increasing numbers of studies examining intracellular microbial metabolites as well [38, 72]. The fecal metabolome is often regarded as an endpoint readout of biological processes originating from the gut microbiome [73]. Identified metabolites in fecal metabolomics can include those derived from the microbiota (e.g., lipopolysaccharide and butyrate) or the host (e.g., anti-microbial peptides). These metabolites can often act as signalling markers that allow for communication between the host and microbiome. In fact, many metabolites in the intestine are produced by co-metabolisms of the host and their microbiome, and intestinal metabolic imbalances have been commonly implicated in disease development [37, 74, 75]. Profiling metabolomes in fecal samples or targeted analysis of drug metabolites during drug treatment can provide valuable information on bi-directional drug-microbiome interactions that may contribute to drug pharmacodynamics, pharmacokinetics, or toxicity.

## Integrative multi-omics for studying the host-microbiome interactions

Integrating the data from multi-omic approaches provides additional insight into microbiome functions. For example, integrating metagenomics and metatranscriptomics enables the calculation of transcript/gene ratios, which is indicative of gene transcriptional activation or repression. Metaproteomics is also frequently integrated with metagenomics for either facilitating protein identification from MS spectra using a matched metagenome database search strategy or for calculating microbiome protein expression [34, 76, 77]. Metabolomics is increasingly integrated with metagenomics for identifying co-variation patterns between metabolites and microbiota composition/function and for characterizing phylogenetic specific contributions to metabolite production [39, 40, 78–81]. An excellent example of an integrative multi-omics study was carried out by Heintz-Buschart et al. [33], who characterized microbiome functions in patients with type 1 diabetes (T1D) using metagenomics, metatranscriptomics, and metaproteomics. Their study identified various differentially abundant microbial transcripts encoded by microbes whose abundance was unresponsive to T1D. In addition, the metaproteomic profiling identified several fecal human proteins that correlated with microbial functional profiles. These findings highlight the usefulness of integrating functional meta-omics approaches for host-microbiome interaction studies.

Unfortunately, integrating multi-omic datasets is not a trivial task due to the increased complexity and diversity of the collected data (e.g., data structure, measurement depth, potential errors, etc.). This integration is increasingly reliant on efficient bioinformatic tools, advanced statistical methods, such as multivariate statistics and machine-learning approaches (readers are directed to the following representative reviews for more details [45–47, 82–84]). Correlation analysis, such as Pearson’s or Spearman’s rank correlation, and correlation-based network analysis are the most straightforward and commonly used approaches for multi-omics data integration. Multivariate statistical methods, such as partial least squares regression, orthogonal partial least squares and nonmetric multidimensional scaling [39–41], have also been applied to identify key features that contribute to the association of two or more –omics data sets. The similarity/correlation between multi-omic datasets can be evaluated using statistical approaches such as Procrustes analysis and multiple co-inertia analysis [40, 78, 80]. A further goal of multi-omics data integration is to generate and validate microbiome metabolic networks/models. Although this is still challenging, promising steps forward have been made, including the generation of > 700 genome-scale metabolic reconstructions [85], the development of tools for microbiome metabolic modeling/prediction [86, 87], and the establishment of inter-species metabolic network databases [88]. Recently, several microbiome studies have also taken advantage of machine learning methods, such as random forest algorithms, to either differentiate between health and disease states or identify features that predict clinical outcomes [89–92]. The application of advanced machine-learning approaches will likely revolutionize our ability to integrate and interpret multi-omics data [93, 94]. These future integrations may include the generation of microbiome-scale metabolic reconstructions, which would further push the frontiers of translational microbiome research.

In summary, although multi-omics data integration is still challenging, the integration of multiple meta-omics datasets provides a promising approach to comprehensively characterize the composition, functional, and metabolic activity of microbiomes. This is of particular importance for microbiome research to be translated into clinical applications. The chronic human diseases, such as T1D, diabetes, or IBD, that are often associated with microbiome alterations, are unlikely to be caused by a single bacterium or a single protein/metabolite. Therefore, we anticipate that meta-omics approaches, along with their decreased costs and increased throughput, will become a first-choice analytical method for microbiome-based clinical or pharmaceutical practice.

## Meta-omics in the study of drug-microbiome interactions

The responses of microbiome to external treatments, such as diet and drugs, are usually dependent on the initial microbiome composition, which is highly variable between individuals. A holistic understanding of the interactions between drugs and microbiomes using meta-omics approaches would be helpful in predicting the outcomes of drug treatment or guiding the usage of drugs. Many clinically prescribed drugs can be metabolized by the gut microbiome and/or modulate gut microbiome composition; these drug-microbiome interactions can thus affect drug efficacy and/or toxicity [17, 95, 96]. Examples of these include antibiotics (which would be expected to modulate the gut microbiome) [97], as well as host-targeting drugs, such as metformin and nonsteroidal anti-inflammatory drugs [14, 96]. A recent study by Maier et al. screened > 1000 marketed drugs against 40 human gut microbial strains and found that 24% of the non-antibiotic drugs could inhibit specific gut bacterial species [16]. Zimmermann et al. also reported that around two-thirds of their selected 271 oral drugs were metabolized by at least one of their 76 cultured human gut bacterial strains [98]. These findings provide further evidence for the widespread existence of drug-microbiome interactions in marketed drugs and the importance of evaluating their effects on entire microbiomes. Unfortunately, the detailed interactions between human gut microbiomes and these drugs are still largely unknown, and fewer than 100 drugs have been recorded in drug-microbiome interaction databases [99]. In addition, the few interactions that are recorded often provide little insight as to whether the drug-microbiome interactions may lead to positive, negligible or even negative outcomes for the host. Therefore, the development of high-throughput platforms to rapidly characterize drug-microbiome interactions is urgently needed. Most previous drug-microbiome interaction studies have been performed using animal models, which are time consuming, expensive and not always representative of what will occur in humans. Ex vivo culturing of entire human microbiomes when combined with meta-omics analysis provides a promising way to develop microbiome assays for rapidly screening drug-microbiome interactions against individual microbiomes.

**Fig. 2:**
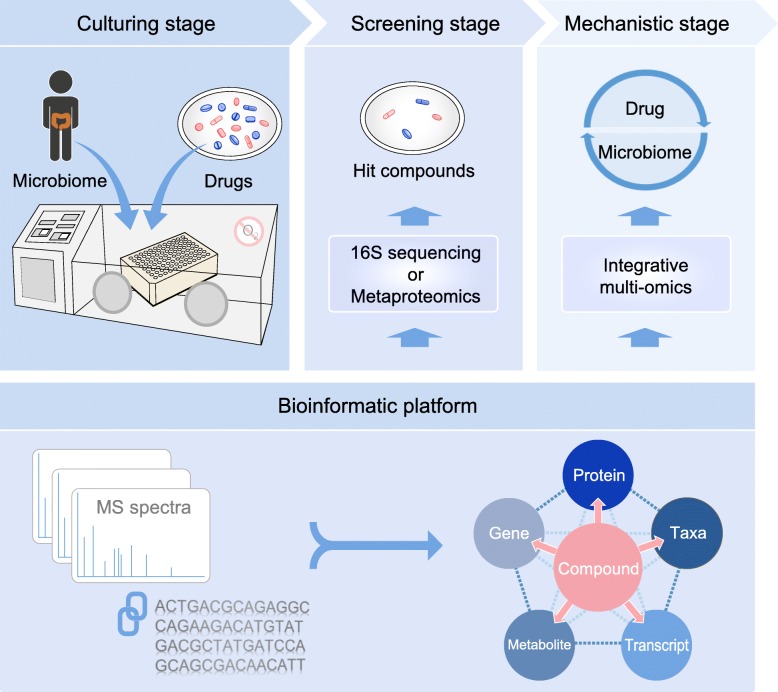
Framework of an ex vivo assay for screening drug-microbiome interactions. The individual’s microbiome is cultured and treated with drugs in anaerobic conditions simulating the in vivo environment. The cultured samples can then be analysed by 16S rRNA gene sequencing or single-shot metaproteomics to rapidly identify hit compounds taking advantage of well-established bioinformatic platforms. Detailed bidirectional drug-microbiome interactions for hit compounds can then be further evaluated with integrative multi-omics approaches

The technology for high-throughput microbiome assays is often adapted from current cell culture-based, host-targeting drug screening platforms. However, there are several challenges inherent to microbiome assays and include (1) the representability of the cultured microbiome, (2) the throughput of microbiome culturing, and (3) the throughput of data generation and processing. Over the past few years, new developments have improved our ability to culture entire human gut microbiota. Lagier et al. reported the culture of > 1000 species from human gut microbiome samples and identified a set of 70 best culture conditions for growing gut microbiota [7]. Fenn et al. utilized a co-culture technique to culture human gut microbiota and identified an essential nutrient (menaquinone), which may help better maintain microbiomes in vitro [100]. Li et al. recently proposed an orthogonal experimental design to rapidly determine key factors in culture media that impact microbiome composition/function and thereby optimize in vitro culture media for specific microbiomes [101]. In addition to static batch culturing systems, microfluidic devices for continuous culturing, such as HuMiX and SHIME [30, 31], have also been developed. Continuous flow devices enable better simulation of in vivo intestinal conditions for the growth of microbiome; however, they are more expensive and cannot be easily adapted for high throughput culturing of many different microbiomes/conditions in a short timeframe. As such, most high throughput screening microbiome assays use batch culturing approaches. Rapid generation of microbiome data using a single –omics approach is now also feasible as technologies and bioinformatic tools for meta-omics analysis are available and being continuously optimized (see above). Multiple –omics approaches can be simultaneously applied to drug-microbiome screening; however, the throughput will be reduced, and costs will be greatly increased. As such, a two-stage approach consisting of an initial rapid screening with a single –omics approach and a second stage consisting of multi-omics characterization for the selected hits is currently more practical to enable high throughput screening and characterization (Fig. 2). Good examples of first step screening tools are 16S rRNA gene sequencing, due to its lower cost, or single-shot metaproteomics given that it provides information on microbiome biomass, composition, and function.

We have recently reported a proof-of-concept high throughput ex vivo microbiome assay, termed rapid assay of individual’s microbiome (RapidAIM) that is based on culturing an individual’s entire microbiome followed by metaproteomic measurement [32]. We showed that RapidAIM maintained microbiome structure and functional profiles for up to 48 hours and recapitulated known in vivo drug effects on microbiomes. We evaluated the responses of individual microbiomes against 43 compounds and found that 27 compounds had significant effects on microbiome composition and function. Chankhamjon et al. adopted a similar microbiome batch culture platform for the rapid screening and detailed characterization of microbiome-derived drug metabolism [102]. Briefly, a healthy microbiome was co-cultured with a library of drugs and the drug metabolites were analysed using HPLC-MS. Among the > 500 oral drugs tested, they discovered that 13% could be metabolized by the microbiome [102]. These studies demonstrate the feasibility of applying high throughput microbiome assays for assessing bi-directional interactions between microbiome and clinically used drugs. The extensive screening of drug-microbiome interactions may also represent an economic way to discover currently approved drugs which have impacts on the microbiome and potentially repurpose these drugs for microbiome-targeted disease therapy.

## Meta-omics at the interface of microbiome and precision medicine

**Fig. 3:**
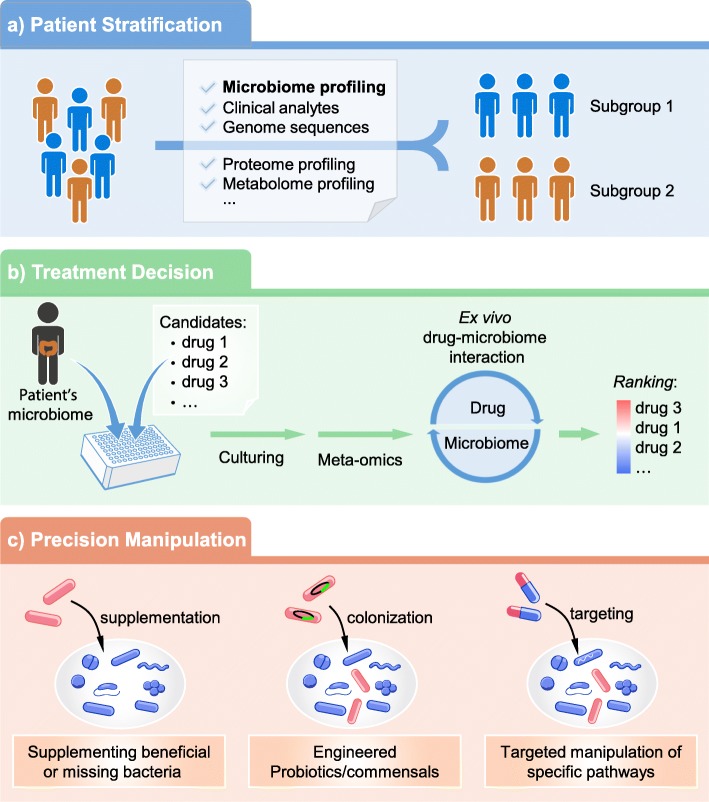
Introducing microbiomes into clinical practice for precision medicine. The profiles of individual patient microbiomes are analyzed with meta-omics, which allow for patients to be classified into sub-groups, i.e., responders vs. non-responders to treatments (**a**). The in vivo response of an individual’s microbiome to drugs can also be predicted with ex vivo microbiome assays, allowing the selection of the best drugs or adjuvant treatments for different patients (**b**). Finally, health and disease management could be carried out by precisely manipulating of the microbiome through supplementing commensal bacteria, engineered bacteria, microbiome-targeted drugs or bacteriophages (**c**)

Precision medicine is an emerging concept for health management given that responses to therapeutic interventions usually vary between individuals. In the past, these variations were assumed to be simply caused by subtle differences between patient genetic backgrounds or due to epigenetic factors controlling host gene expression. For example, genomics-based precision medicine has often been applied in cancer therapy [103–106]. However, it should be noted that many cancer therapeutics could also alter the gut microbiome [16, 107, 108]. More recently, variations in patient responses to cancer immuno- and chemo-therapies were linked to inter-individual differences in gut microbiomes [74, 109–114]. These findings suggest an opportunity to further optimize disease therapies through microbiome-informed patient stratification, through personalized treatment decisions and/or through direct manipulation of patient microbiomes (Fig. 3). They also highlight the importance of including microbiomes into the framework of precision medicine [19].

### Patient stratification based on microbiome profiling

One important goal of precision medicine is to identify biomarkers for stratifying patients into subgroups that are likely to be responsive (or unresponsive) to a given treatment [115]. As mentioned above, heterogeneous responses of patients to treatment may be due in part to differences in their gut microbiomes. Therefore, a prior understanding of an individual’s microbiome may help predict treatment outcomes and/or suggest optimal therapeutic strategies (Fig. 3a). Gu et al. demonstrated that the gut microbiome of new-onset type 2 diabetes (T2D) patients could be classified into two clusters, namely cluster P (dominated by *Prevotella*) and B (dominated by *Bacteroides*), and found that cluster P patients had greater metabolic improvement after 3-month acarbose treatment as compared to cluster B patients [116]. In a prospective wellness study, Price et al. [117] illustrated the use of dense and dynamic personal data clouds, including host genetic traits, clinical analytes, metabolites, proteomes, and microbiomes, to identify candidate markers for predicting the transition from health to disease. In the disease-prone subgroup, life style interventions stemming from these personalized-data biomarkers successfully improved their health status [117]. In cancer therapy, immune checkpoint inhibitors targeting the programmed death 1 (PD-1) protein are important therapeutics but are only effective in a subset of patients. Recent studies have shown that patient’s failure to respond to anti-PD-1 therapy could be attributed to the absence or under-representation of certain immune-regulating bacterial species in gut, namely *Akkermansia muciniphila*, *Faecalibacterium*, and *Bifidobacterium longum* [109–111]. These findings suggest that quantifying these commensals in patient fecal samples may help predict therapeutic outcomes and stratify patients into potential responders or non-responders to PD-1 blockade.

### Ex vivo microbiome assays for guiding treatment decisions

The gut microbiota is a highly diverse microbial community with high inter-individual variability, making in vivo drug-microbiome interactions complex and an individual’s response to drug treatment difficult to predict. In addition, as mentioned above, there is no knowledge on drug-microbiome interactions for the majority of clinically prescribed drugs. A prior understanding or prediction of drug-microbiome interactions in a patient through microbiome assays would be invaluable for optimizing the therapeutic outcomes in diseases that are known to be associated with gut microbial alterations. This would allow for patients to be prescribed the most effective drug for treating their disease (Fig. 3b). For example, digoxin is a commonly used cardiac drug and can be converted into its inactive form, dihydrodigoxin, by specific strains of the intestinal bacterium *Eggerthella lenta*, and this has been suggested to contribute to digoxin’s varied bioavailability among individuals [118, 119]. However, the extent of digoxin inactivation is also dependent on the presence of other gut microbes [118], indicating that a single biomarker using the presence of *E. lenta* species may not be sufficient for patient stratification. Instead, culture of digoxin with an ex vivo microbiome followed by metabolite measurement can more accurately predict the extent of digoxin inactivation and thereby guide the decision on whether adjuvant intervention, such as arginine supplements or antibiotics, is needed [118, 120]. In addition, for diseases with multiple drug candidates, such as IBD [121], culturing a set of candidate drugs with an individual’s ex vivo microbiome may help select the most likely effective drug candidate for treating each patient’s disease.

### Targeted manipulation of microbiome for precision disease treatment

Although the gut microbiome has long been considered as a potential target for disease treatment [13] and an effective microbiome-targeted dietary intervention approach has been demonstrated [9], commercially available targeted therapeutics for precise modulation of microbiomes are still lacking. However, our understanding of the mechanisms underlying host-microbiome interactions is growing rapidly, and new potential targets (e.g., specific microbial species or metabolic pathways) in the microbiome are being identified. It may soon be feasible to precisely manipulate the microbiome through either supplementation of beneficial species (such as *A. muciniphila*) [111], engineered probiotics/commensals [122], prebiotics [9], bacteriophages [123], or highly selective drugs [124] (Fig. 3c). For example, Zhu et al. recently reported that tungstate can specifically inhibit molybdenum-cofactor-dependent gut microbial respiratory pathways under inflammatory conditions, which ameliorates intestinal colitis and restores gut microbial homeostasis in a mouse model of colitis [124]. More recently, Ho et al. reported that a genetically modified *E. coli* strain, which has selective affinity to cancer cells and secrets myrosinase for converting vegetable derived glucosinolate into anti-cancer compounds, effectively prevented the development of cancer in mice receiving a cruciferous vegetable diet [125]. Dietary intervention is another safe and promising approach for manipulating the microbiome. Zhao et al. utilized a specialized diet to promote the growth of a group of short-chain fatty acid-producing bacteria in the gut of T2D patients, which was proposed to have contributed to improved glucose homeostasis in these patients [9]. Along with the development of sophisticated tools for manipulating microbial genetics [122, 126], it is becoming feasible for targeted modulation of specific microbial metabolic pathways or species in microbiome, which further lays the foundation for future microbiome-targeted therapies.

## Conclusions

The ultimate goal of human microbiome research is to facilitate health and disease management. Gut microbiome alterations have been associated with an increasing list of diseases, and selectively modifying the gut microbiota has been shown to alleviate the development of disease, including diabetes and colitis. These achievements highlight the importance of introducing the microbiome into the precision medicine framework, through either microbiome-guided patient stratification or interventions that specifically target microbial species/pathways. However, it is still a challenge to rapidly identify specific, actionable targets within microbiomes. Fortunately, the addition of metatranscriptomics, metaproteomics, and metabolomics to metagenomics is enhancing our functional understanding of the microbiome. Although more powerful and convenient bioinformatic tools are still needed, integrative functional meta-omics is becoming one of the most important approaches for dissecting microbial metabolic pathways in microbiomes. In addition, the development of microbiome-targeted drugs is also challenging. Therefore, efforts are underway to develop new ex vivo assays targeting panels of individual bacteria, simple microbial communities, or entire microbiomes. These are likely to rapidly increase our understanding of how microbiomes interact with drugs, food components, and natural compounds. Ex vivo microbiome assays will likely be useful in precision medicine by allowing individual microbiomes to be screened against panels of drugs/compounds to select the most efficient treatment.

## Data Availability

Not applicable

## Abbreviation

IBD: Inflammatory bowel disease
MS: Mass spectrometry
NMR: Nuclear magnetic resonance
T1D: Type 1 diabetes
T2D: Type 2 diabetes
PD-1: Programmed death 1
